# Surface Activation of Pt Nanoparticles Synthesised by “Hot Injection” in the Presence of Oleylamine

**DOI:** 10.1002/chem.201501496

**Published:** 2015-07-17

**Authors:** Jo J L Humphrey, Sajanikumari Sadasivan, Daniela Plana, Verónica Celorrio, Robert A Tooze, David J Fermín

**Affiliations:** a School of Chemistry University of Bristol, Cantocks Close, Bristol, BS8 1TS (UK), Fax: (+44) 117-927-7985; b Sasol Technology UK Ltd. Purdie Building North Haugh, St Andrews, Fife, Scotland, KY16 9ST (UK)

**Keywords:** electrocatalysis, nanoparticles, platinum, supported catalysts, surface activation

## Abstract

Oleylamine (OA) based “hot injection” colloidal synthesis offers a versatile approach to the synthesis of highly monodisperse metallic and multi-metallic alloyed nanostructures in the absence of potentially toxic and unstable phosphine compounds. For application in heterogeneous catalysis and electrocatalysis, the adsorbed OA species at the metal surfaces should be effectively removed without compromising the structure and composition of the nanostructures. Herein, we investigate the removal of OA from colloidal Pt nanoparticles through 1) “chemical methods” such as washing in acetic acid or ethanol, and ligand exchange with pyridine; and 2) thermal pre-treatment between 185 and 400 °C in air, H_2_ or Ar atmospheres. The electrochemical reactivity of Pt nanoparticles is acutely affected by the presence of surface organic impurities, making this material ideal for monitoring the effectiveness of OA removal. The results showed that thermal treatment in Ar at temperatures above 400 °C provides highly active particles, with reactivity comparable to the benchmark commercial catalyst, Pt/ETEK. The mechanism involved in thermal desorption of OA was also investigated by thermogravimetric analysis coupled to mass spectrometry (TGA-MS). Oxidation of HCOOH and adsorbed CO in acidic solution were used as test reactions to assess the Pt electrocatalytic activity.

## Introduction

Electrocatalysts typically consist of a catalytically active component, such as metallic nanoparticles, incorporated into a support material, often carbon (mesoporous, nanotubes, graphene, diamond)^1^, ^2^ or metal oxides (e.g., WO_3_, TiO_2_).^3^ There are two general preparation routes of these composite materials: impregnation^4^, ^5^ and colloidal synthesis.^6^–^8^ In the impregnation approach, metal clusters can be nucleated directly on the support by reduction of metal salts in the presence of strong electron donors such as NaBH_4_.^4^ Though this one-step process is straightforward and enables high throughput of electrocatalyst material, there is little control over the size and elemental composition of the nanoparticles, which can lead to difficulties when establishing structure–activity relationships, and controlling catalytic activity or selectivity.

In the colloidal synthetic approach, nanoparticles are first synthesised, before subsequently being deposited onto the required support.^6^–^8^ Although this is a two-step procedure, it allows for more precise control over particle size and metal ratios than the impregnation method described previously, therefore enabling catalyst structure–activity relationships or catalyst-support interactions to be identified.^2^, ^9^, ^10^ Colloidal thermolytic synthesis can be performed by the “heat up” or “hot injection” approaches.^11^, ^12^ In the former case, the reaction mixture is heated, leading to reduction of the precursors at higher temperature, whereas in “hot injection” the cold solution of precursors is injected at higher temperatures into the hot reaction mixture.^11^, ^12^ This solution-phase synthesis is employed in the preparation of a wide range of materials, including semiconductors, quantum dots, mono- and multimetallics, magnetic particles and metal oxides, all at the nanoscale and with control over their size, shape and composition.^11^, ^12^ Oleylamine (OA) is a versatile high boiling point solvent used in the so-called “phosphine-free hot-injection synthesis”, and also plays the role of reducing agent at higher temperatures.^11^, ^12^ In the case of metal nanoparticles, it is able to co-ordinate through lone-pair donation from the nitrogen atom of the amine group, promoting steric stabilisation of the nanoparticles.^12^ The removal of these surface groups, without compromising the structure and composition of the nanomaterial, is a crucial step in areas such as heterogeneous catalysis, opto-electronic devices, sensors and functional optical probes.

Numerous methods exist for the removal of capping agents from the surface of nanoparticles; the specific procedure is often dependent on the nature of the nanomaterials, as well as the particular surfactant.^6^, ^13^–^16^ For shaped nanoparticles or single crystal surfaces, removal of surfactants is typically performed by a solvent washing procedure in order to maintain the morphology. For example, polyvinyl(pyrrolidone), PVP, can be removed from Pt(111) by centrifugation in H_2_SO_4_:H_2_O_2_, without disturbing the superficial order.^13^ Alternatively, this common capping agent can be removed by centrifugation in ethanol and *n*-hexane mixtures.^14^ However, fully stripping this stabiliser from the colloidal nanoparticles can lead to aggregation and self-assembly phenomena, which may impact on electrocatalytic properties.^14^ In other examples, citrate and polyacrylate can be removed by centrifugation in NaOH,^15^ and polyvinyl alcohol capped Au particles can be cleaned by simply refluxing in water.^16^

Thermal annealing procedures are also common, although these may lead to changes in particle size and morphology, particularly at high temperatures. Long et al. showed that heating PVP-capped polyhedral Pt nanoparticles at 300 °C caused a significant change in morphology, and the nanoparticles had a lower electrocatalytic activity than if the nanoparticles were centrifuged in *n*-hexane/ethanol prior to annealing.^14^ Meanwhile, the groups of Nikles and Markovic both stated that heat treatment of oleylamine-capped platinum nanoparticles at 185 °C in air was sufficient to activate them for electro- oxidation reactions, whilst inducing minimal changes in particle size.^6^, ^17^

In this paper, various methods are investigated for the removal of OA from nanocrystalline Pt, as obtained by “hot-injection” colloidal synthesis, with the aim of critically assessing protocols for surface activation. The efficacies of several chemical and thermal pre-treatment methods were assessed in terms of changes to the nanoparticle surface and the impact of their electrocatalytic properties with regards to HCOOH and surface-adsorbed CO (CO_ads_) oxidation in acidic solution. It is shown that thermal annealing procedures in H_2_ or Ar atmospheres at moderate temperatures (300–400 °C) are highly effective for removing long chain OA, leading to highly active electrocatalysts. The processes involved in the thermal pre-treatment are investigated by thermal gravimetric analysis coupled to mass spectrometry (TGA-MS). We conclude that some of the most effective protocols for OA removal can be extended to a wide range of materials, including chalcogenide quantum dots, without compromising their size and composition.

## Results and Discussion

### Electrocatalyst synthesis and characterisation

**Figure 1:**
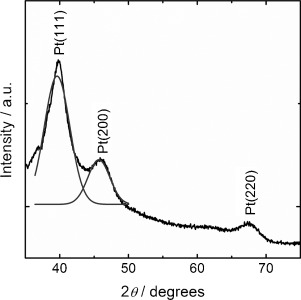
X-ray diffractogram of as-prepared Pt nanoparticles, also indicating the deconvoluted signals of the Pt(111) and Pt(200) peaks, used for Scherrer analysis of particle size.

Figure 1 shows a typical powder XRD pattern of dried colloidal Pt nanoparticles, as prepared. Peaks corresponding to Pt(111), Pt(200) and Pt(220) are seen at 39.6°, 45.7° and 67.3°. The broad signals were deconvoluted, and subsequent Scherrer analysis gave an average crystallite size of 3.4 nm.

**Figure 2:**
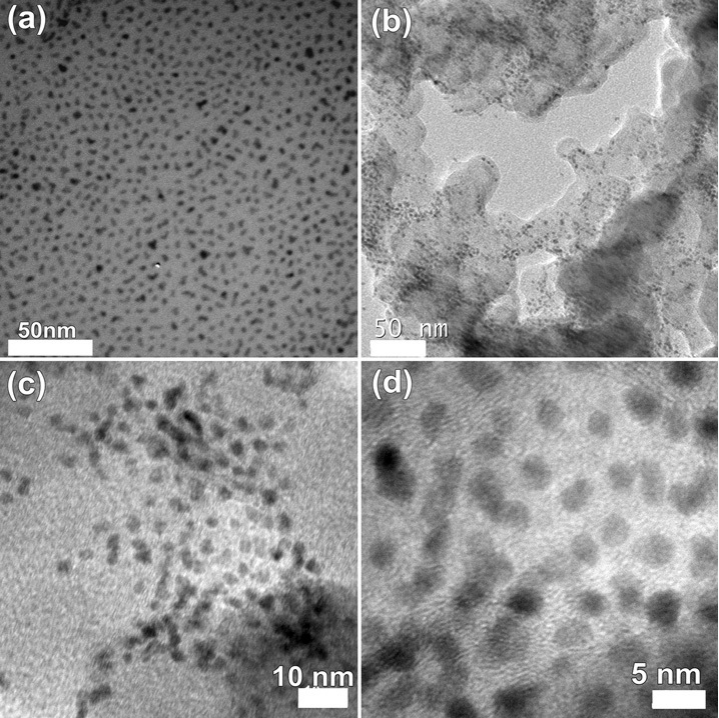
a) TEM of as-prepared Pt nanoparticles; b) TEM image of Pt/C catalyst; c) and d) HR-TEM images of as-prepared Pt/C catalyst.

The high level of monodispersity of the as-prepared particles is demonstrated by the TEM images in Figure 2. Contrasting the images of as prepared Pt nanoparticles (Figure 2 a) and Pt/C (Figure 2 b–d) reveals that the structural integrity of the nanomaterial is not affected, in particular size distribution, during the incorporation into the mesoporous carbon support. Analysis of the TEM micrographs resulted in average size of 3.5±0.2 nm, in excellent agreement with the value obtained by use of the Scherrer equation in conjunction with the powder XRD pattern (Figure 1).

**Figure 3:**
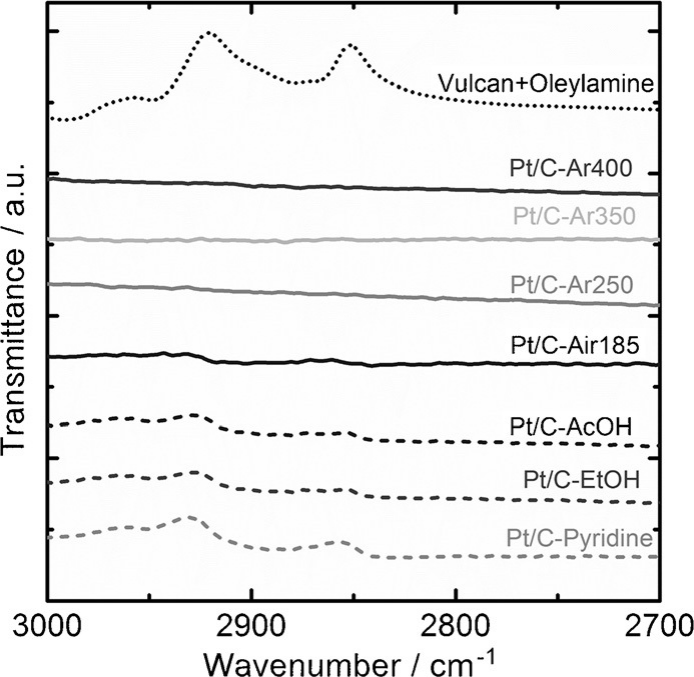
FTIR spectra of various Pt/C electrocatalysts between wavenumbers 3000–2700 cm^−1^. For comparison, spectra of OA adsorbed on Vulcan, “Vulcan+oleylamine”, is also included.

Figure 3 shows the FTIR spectra of the Pt/C electrocatalysts between 3000–2700 cm^−1^ as a function of the various chemical and physical pre-treatment protocols. These spectra are also compared to OA adsorbed on Vulcan (Vulcan+oleylamine). The removal of OA is confirmed by the disappearance of the bands at 2920 and 2850 cm^−1^, corresponding to its CH_2_ asymmetric and symmetric stretching modes, respectively.^6^, ^12^ For all of the chemical treatments studied, signals for these stretching modes of OA are still present in the IR spectra, indicating that these methods were not fully effective in removing the capping agent from the electrocatalysts. The Pt/C-Air185 sample also showed small signals at 2920 and 2850 cm^−1^ in the IR spectra, indicating that the catalyst surface was not fully cleaned by this procedure. In oxygen-free conditions, higher annealing temperatures were required in order to effectively remove OA. Results indicated that thermal approaches in Ar atmosphere were the most effective methods for obtaining ligand free nanoparticles, as no bands related to OA were observed in the IR spectra. In order to confirm that the remaining OA is adsorbed either on the Pt nanoparticles or the carbon support, electrochemical studies involving surface sensitive probes are required.

### Pt activation as probed by HCOOH oxidation

**Figure 4:**
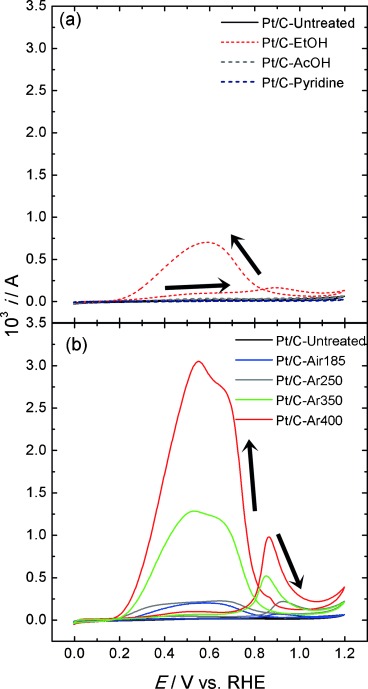
Cyclic voltammograms of formic acid oxidation for a) chemically pre-treated, and b) thermally pre-treated Pt/C electrocatalysts, recorded in 0.5 m H_2_SO_4_ and 2.0 m HCOOH solution, at a scan rate of 0.020 V s^−1^.

Figure 4 illustrates cyclic voltammograms of the Pt/C electrocatalysts prior to and after the various pre-treatments in sulfuric acid solutions containing HCOOH at 0.020 V s^−1^. The initial potential was set at 0.0 V, followed by scanning to 1.2 V and back to the initial potential. For these studies, metal loading was kept low (2.24 wt %), which provides high enough currents to illustrate the contrast in reactivity in initial screening experiments.

Pt is a highly active electrocatalyst for formic acid oxidation, showing rather complex features originating from the generation of intermediate species such as adsorbed formate and CO.^18^, ^19^ A detailed discussion on the various voltammetric features is beyond the scope of this work. In order to assess electrocatalytic activity of the various Pt/C electrocatalysts, the peak current densities in the backward scan (between 0.5–0.6 V) are compared.

Figure 4 shows that the as-prepared Pt/C catalyst shows no activity towards HCOOH, even after potential cycling, as surface sites are blocked by OA ligands.^6^, ^17^, ^20^ It is evident that the thermal methods investigated were in general more effective at removing the OA surfactant than the chemical methods. Of the chemical methods studied here, only the centrifugation in ethanol led to a catalyst (Pt/C-EtOH) that demonstrated activity towards HCOOH oxidation. On the other hand, all thermal methods investigated showed improvement in the oxidation current density compared to untreated Pt/C.

Pt/C-Air185 showed very low catalytic reactivity towards HCOOH oxidation. This is in contrast to previous reports, which have indicated that heating to 185 °C in air was sufficient for obtaining a clean, active catalyst for methanol oxidation.^17^ This may be related to the fact that the annealing temperature was significantly lower than 364 °C, the boiling point of liquid OA. Similar results are obtained for Pt/C-Ar250 and Pt/C-Ar350, where the peak current densities are lower than that observed for Pt/C-Ar400. It is also important to consider that the thermal pre-treatment can affect the average particle size. As discussed further below, the average size of Pt/C-Ar400 increased to 6.0±2.6 nm. Previous studies have shown size-dependent reactivity of Pt with sizes below 5 nm.^17^, ^21^, ^22^

**Figure 5:**
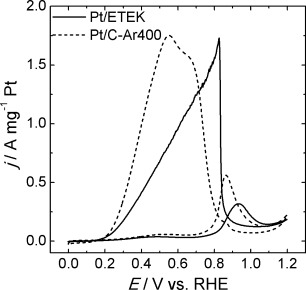
Cyclic voltammograms of formic acid oxidation with Pt/C-Ar400 and Pt/ETEK catalysts. Experiments were carried out in 0.5 m H_2_SO_4_ and 2.0 m HCOOH at a scan rate of 0.020 V s^−1^.

Figure 5 contrasts the electrocatalytic activity of Pt/C-Ar400 with the commercially available Pt/ETEK, a benchmark electrocatalyst for formic acid oxidation. Pt/ETEK features average nanoparticles sizes of 4.1±0.8 nm, incorporated into a mesoporous carbon support with a metal loading of 17.5 wt % Pt. It is interesting to see that the peak current densities of the two catalysts are very similar, confirming a highly active Pt surface. Differences in the shape of the voltammograms can be linked to the difference in overall metal loading, leading to substantially higher currents for the Pt/ETEK electrocatalysts.

Analysis of the catalyst activity, based on HCOOH oxidation, clearly shows that thermal pre-treatment in Ar with temperatures above 350 °C provides an optimum methodology for surface activation. Similar conclusions can be reached by estimating the electrochemical surface active area by oxidation of adsorbed CO (CO_ads_), as discussed in the Supporting Information. The next section will analyse the processes behind the thermal surface activation.

### TGA-MS study of OA removal from Pt/C

**Figure 6:**
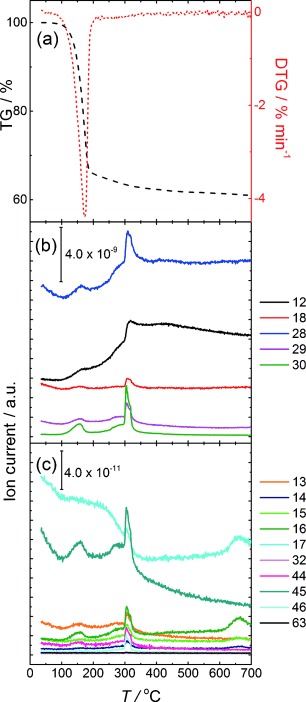
a) TG and DTG curves; b) and c) corresponding MS plots for Pt/C electrocatalyst, heated to 700 °C in 10 % H_2_ in argon. Legend values correspond to *m/z* of fragments monitored during TGA.

Figure 6 a shows the TG curve and its first derivative (DTG) under H_2_/Ar of Pt/C (15 wt % metal loading). The data show a weight loss of 34 wt % in the temperature range of 100 to 200 °C. However, the corresponding MS showed only minor features corresponding to desorption of hydrocarbon fractions CH_3_CH_2_^+^ with *m/z* values of 29, 28 and 15. A further steady decrease in mass is also observed up to around 350 °C. The monitored *m/z* fragments are characterised by a sharp peak in the ion current between approximately 300–330 °C. Most of these MS signals correlate to a range of C_1_–C_3_ hydrocarbon fragments, including amine fragments C_1_NH_2_^+^ and C_2_NH_2_^+^. Particularly interesting are the *m/z* fragments 18 and 45, which correspond to H_2_O and C_2_NH_7_^+^, respectively. The H_2_O signal at such a high temperature is unusual, and is attributed to loss of water from oxygenated surface functionalities of Vulcan. Furthermore, the detection of the C_2_NH_7_^+^ ion may indicate that degradation of the amine is occurring at the Pt surface, rather than in the gas phase. These results are in line with previously reported TGA data for OA-stabilised Pt nanostructures,^6^, ^8^, ^17^ although analysis of the fragments released as a function of temperature has not been reported so far.

The TGA-MS data suggest that the thermal OA desorption from Pt/C catalysts occurs in two stages. The first stage involves cleavage of the C=C bond of OA into fragments of various sizes, which desorb below 200 °C. Residual longer chain fragments later decompose on the catalyst surface at around 300–330 °C, generating smaller hydrocarbon fragments as detected by MS. Finally, the remaining slow decrease in the sample mass up to 700 °C is attributed to degradation of carbon support, which may take place at lower than expected temperatures due to the influence of incorporated Pt nanoparticles.^19^, ^23^

### Thermal activation: H_2_ versus Ar atmospheres

**Figure 7:**
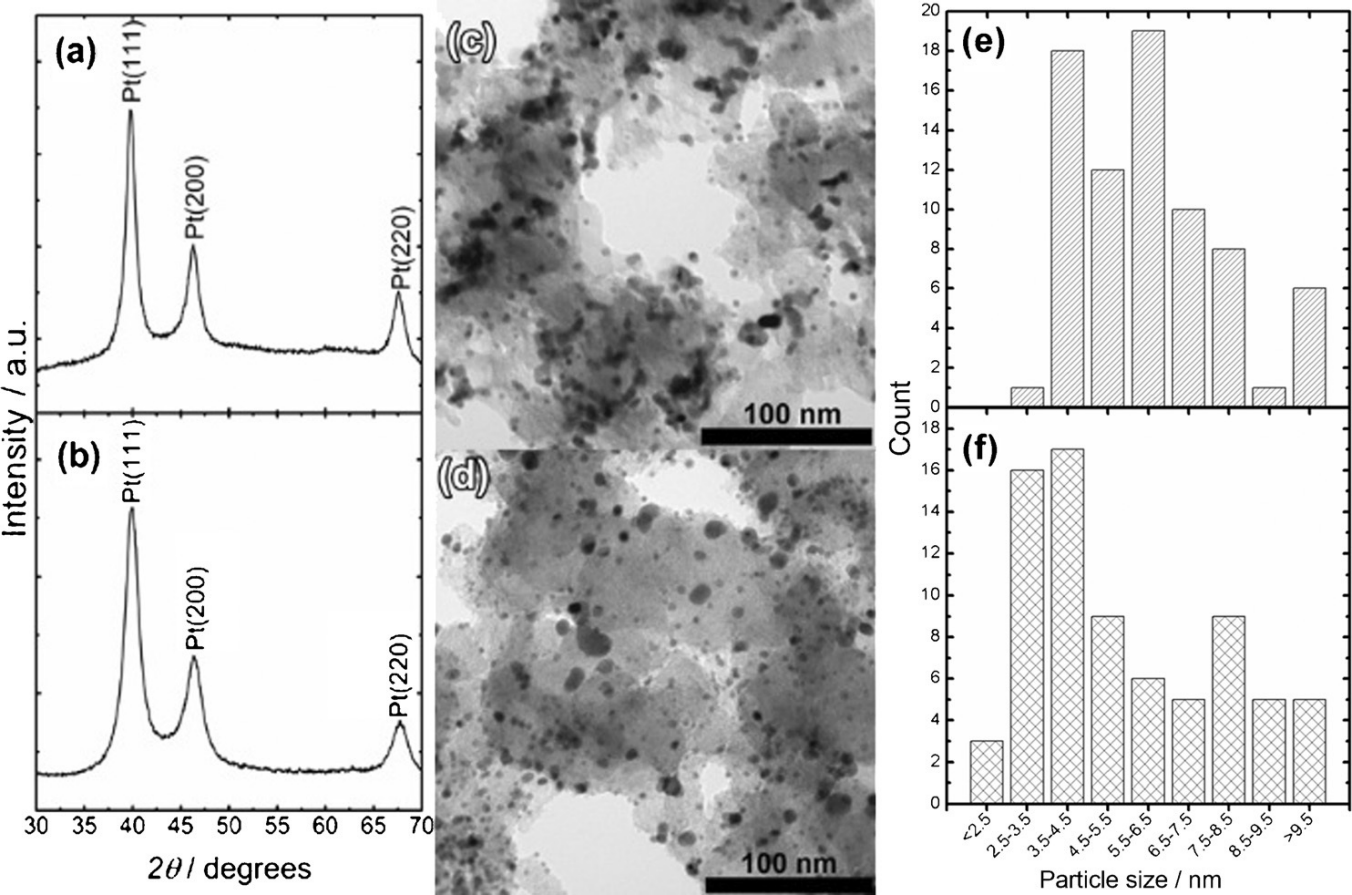
Powder XRD patterns of a) Pt/C-Ar400 and b) Pt/C-H_2_300 electrocatalysts after thermal activation; TEM images of c) Pt/C-Ar400 and d) Pt/C-H_2_300; histograms of primary particle size for e) Pt/C-Ar400 and f) Pt/C-H_2_300.

Figure 7 contrasts powder XRD patterns, TEM micrographs and particle size distribution of Pt/C thermally pre-treated at 400 °C under Ar (Pt/C-Ar400) and at 300 °C under H_2_ (Pt/C-H_2_300). It should be clarified that the main criterion for selecting the pre-treatment temperature for both atmospheres mainly relies on TGA analysis. In other words, we have selected the lowest temperature at which OA removal is completed as probed by TGA. As discussed at the end, this issue is important when considering pre-treatment of OA established nanostructures whose composition can be affected by the presence of H_2_ at elevated temperatures. In this case, statistical analysis of the TEM data suggests a slightly larger mean particle diameter for Pt/C-Ar400 (6.0±2.6 nm) than Pt/C-H_2_300 (5.5±1.9 nm). Both pre-treatments lead to the formation of aggregates in the range of 21.7±6.4 nm for Pt/C-Ar400, while Pt/C-H_2_300 features fewer agglomerates of 14.0±3.0 nm.

**Figure 8:**
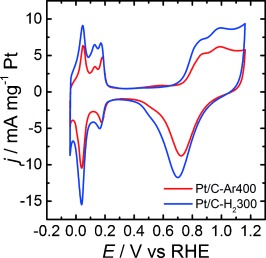
Cyclic voltammograms of Pt/C-H_2_300 and Pt/C-Ar400 electrocatalysts in 0.5 m H_2_SO_4_ recorded at 0.020 V s^−1^.

Cyclic voltammograms of Pt/C-Ar400 and Pt/C-H_2_300 in sulfuric acid solutions at 0.020 V s^−1^ are contrasted in Figure 8. Both sets of materials show extremely clear H adsorption and desorption peaks in the range of 0.2 to 0.0 V, as well as current responses linked to surface Pt oxide of similar magnitude. These features are consistent with highly clean polycrystalline Pt surfaces.^24^ An interesting point is that current responses normalised by the Pt mass are larger for Pt/C-H_2_300 than Pt/C-Ar400. A close examination of the voltammograms in the range of 0.4 to 0.2 V reveals that the double-layer capacitance is rather similar for both samples. This capacitive component is mainly dominated by the high surface area of the mesoporous carbon support, indicating that the difference in the current magnitude of the Pt signatures is not related to an overall larger amount of the Pt/C. The larger Pt voltammetric responses in Pt/C-H_2_300 essentially show a doubling of the effective electrochemically active surface area (ECSA). The origin of this effect could not only be linked to the increase in the mean Pt size and the presence of larger aggregates in Pt/C-Ar400 but also to changes in the conducting properties of the mesoporous carbon as a result of the thermal pre-treatment under H_2_. Lazaro et al. have shown that the electronic conductivity of highly porous carbon films can be affected by the presence of oxygenated groups.^25^ An increase in the effective conductivity, as a result of partial carbon hydrogenation, will lead to a lower population of Pt nanoparticles electrically disconnected from the glassy carbon electrode.

**Figure 9:**
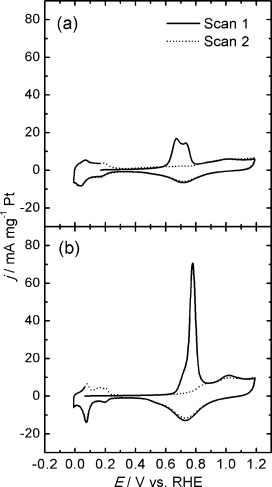
Voltammograms of electro-oxidation of adsorbed CO on a) Pt/C-Ar400 and b) Pt/C-H_2_300 electrocatalysts. Two consecutive scans were recorded at 0.020 V s^−1^ in 0.5 m H_2_SO_4_ following electrosorption of CO onto the surface of the electrocatalysts.

The Pt ECSA can also be approached by examining the voltammetric responses associated with the oxidation of adsorbed CO (CO_ads_), as illustrated in Figure 9. These experiments were carried out by bubbling CO through the solution and holding the electrode potential at 0.03 V for 10 min and subsequently purging with Ar for 20 min to remove any dissolved CO. Two consecutive cyclic voltammograms were recorded, with the first one exhibiting a response between 0.6–0.8 V, linked to the oxidation of CO_ads_ to CO_2_.^24^, ^26^, ^27^ The excess charge associated with CO_ads_ oxidation can be used to estimate the ECSA of Pt, taking the charge density associated with the oxidation of a CO monolayer in polycrystalline Pt as *Q*_CO_=420 μC cm^−2^.^10^ This analysis resulted in ECSA areas of 199 and 427 cm^2^ mg^−1^ for Pt/C-Ar400 and Pt/C-H_2_300, respectively. These figures reflect the larger particle size and, more importantly, the higher degree of particle sintering and agglomeration, for Pt/C-Ar400. We have used this approach to systematically investigate all of the thermal and chemical pre-treatments highlighted in Table S1 in the Supporting Information.

Considering the mobility of CO_ads_ at the Pt surface, the shape of the oxidation response can also provide additional information about the nanostructure of the metal domains. Pt/C-H_2_300 exhibits a sharp CO_ads_ oxidation peak at 0.78 V, with a small shoulder at approximately 0.7 V (Figure 9 b), whereas Pt/C-Ar400 features a broader double peak at 0.62 and 0.72 V (Figure 9 a). The more positive oxidation potential of CO_ads_ on Pt/C-H_2_300 can be linked to the smaller mean crystallite size and lower population of aggregates compare to Pt/C-Ar400. The size dependence and the effect of particle aggregates on the voltammetric responses of CO_ads_ oxidation have been widely investigated in the literature.^17^, ^24^, ^27^–^29^ There is a degree of consensus on the shift of the CO_ads_ oxidation peak towards more positive potential as the particle size decreases, although it remains as a matter of debate whether this trend is due to changes in the Pt–CO or the Pt–OH interaction.^17^, ^21^ The same argument can be put forward for the double peak observed in the voltammogram of Pt/C-Ar400.

## Conclusion

A systematic study of chemical and thermal pre-treatment of OA-capped Pt nanoparticles synthesised by colloidal “hot-injection” reveal that the latter is significantly more effective in removing the capping layer. This conclusion is based on qualitative analysis of the carbon-supported Pt particles employing FTIR and quantitative electrochemical studies using surface-sensitive probes such as H-adsorption/desorption as well as CO_ads_ and HCOOH oxidation. Using the same starting material, we demonstrate that the ECSA can vary from essentially 0 (as-deposited and pyridine washed Pt/C) to approximately 200 cm^2^ mg^−1^ of Pt (Pt/C-Ar400), or 427 cm^2^ mg^−1^ for Pt/C-H_2_300. The latter samples exhibit electrocatalytic properties for HCOOH oxidation comparable with commercial Pt/ETEK. TGA-MS analysis shows that hydrocarbon fragments associated with the C_18_ chain occurs at temperatures below 220 °C. Further fragmentation and desorption of the long-chain amine occurs at a much higher temperature (≈330 °C), because it appears that fragmentation of the amine occurs on the surface of the nanoparticles, rather than in the gas phase.

Finally, the electrochemical performance of Pt/C thermally pre-treated in H_2_ atmosphere at 300 °C was compared to Ar at 400 °C. The two different temperatures correspond to conditions in which complete removal of OA is achieved, as probed by TGA. The electrochemical signatures in sulfuric acid confirm a high degree of OA removal from the Pt surface. However, the average crystallite size Pt/C-Ar400 was 9 % larger than that obtained for Pt/C-H_2_300 with 54 % lower electrochemical surface active area, as determined by H-adsorption and oxidation of adsorbed CO. The larger electroactive surface area could be explained in terms of an increase in the conductivity of the mesoporous carbon as a result of the H_2_ pre-treatment (leading to a higher population of electrically accessible nanoparticles) and a lower degree of Pt aggregation in comparison to the Ar treatment. The broader CO_ads_ oxidation peak shape observed for Pt/C-Ar400 appears consistent with the presence of larger aggregates.

Although the present analysis focuses on Pt nanostructures, our conclusions are relevant to the large range of materials that can be synthesised by “hot injection” in the presence of OA. Indeed, the monodispersity of metal nanostructures capable of tolerating H_2_ thermal treatment would be less compromised by the lower temperatures required under these conditions. On the other hand, the stability of materials such as metal oxides may not be compatible with the presence of H_2_, requiring higher temperatures above 400 °C in either Ar or O_2_ environments. As demonstrated in previous studies,^30^ the acute sensitivity of small Pt nanoparticles to the presence of adsorbed organic species as well as aggregates provide a unique approach to study effective protocols for removal of colloid stabilisers.

## Experimental Section

### Synthesis and pre-treatment of carbon-supported Pt nanoparticles (Pt/C)

Pt nanoparticle synthesis was performed according to the protocol reported by Liu et al.^17^ Briefly, the synthesis is initiated by dissolving 0.25 mmol Pt(acac)_2_ (Acros Organic) and 1.50 mmol 1,2-tetradecanediol (90 %, Sigma Aldrich) in 20 mL dioctyl ether (99 %, Sigma Aldrich) under Ar atmosphere, followed by heating to 110 °C at a rate of 5 °C min^−1^ under stirring. OA (4.2 mmol; 70 %, Sigma Aldrich) was added drop-wise to the clear, yellow solution over the course of 2 min. The solution was further heated to 215 °C, and left to stir for 1 h before cooling to 50 °C. The dark brown particle dispersion was added to a glass vial containing 10 mL MeOH, and sonicated for 5 min. Nanoparticles were precipitated by centrifugation, and re-dispersed in hexane. All high-purity reagents were used as received. To prepare the carbon-supported catalyst (Pt/C), Vulcan XC-72R (Cabot Corp) was added to a colloidal Pt nanoparticle dispersion, and sonicated for 2 h. The solution turned clear, indicating complete deposition of nanoparticles onto the carbon support. Hexane was removed under a stream of argon to yield the dry Pt/C catalyst powder.

A variety of chemical and thermal pre-treatment protocols were investigated for the as-prepared Pt/C nanostructures, as summarised in Table 1 and Table 2. A fixed amount of the Pt/C catalyst (5 mg) was used for each chemical pre-treatment.

**Table 1:** Chemical pre-treatment protocols for Pt/C electrocatalyst.

Sample	Solvent	Conditions
Pt/C-EtOH	ethanol, 10 mL	three times centrifugation at 10 000 rpm
		dry under Ar
Pt/C-AcOH	acetic acid, 15 mL	reflux 6 h, 75 °C
		dry under Ar
Pt/C-pyridine	pyridine, 10 mL	reflux 18 h, 130 °C
		dry under Ar

**Table 2:** Thermal pre-treatment conditions for Pt/C electrocatalyst powder.

Sample	Temperature [°C]	Atmosphere	Time [h]	Ramp [°C min^−1^]
Pt/C-Air185	185	air	5	4.5
Pt/C-H_2_300	300	H_2_ (100 %)	2	5.0
Pt/C-Ar250	250	Ar	5	1.0
Pt/C-Ar350	350	Ar	2	5.0
Pt/C-Ar400	400	Ar	2	5.0

### Catalyst characterisation

X-ray diffraction (XRD) patterns were measured at room temperature on a Bruker AXS Advance D8 diffractometer, using Cu_Kα_ radiation (40 kV, 0.040 A, *λ*=1.5406 Å). Pt nanoparticles or Pt/C suspension were drop-cast onto a Si wafer, and the solvent was left to evaporate at room temperature. Diffractograms were recorded in a 2*θ* range 20–75° (step size 0.05°, step time 30 s). OriginLab 9.0 software was used to deconvolute broad peaks for Scherrer analysis to determine crystallite size.

Transmission electron microscopy (TEM) was performed on a JEOL JEM 1200 EX MKI, and high resolution TEM (HR-TEM) was performed on JEOL 200 Kv Hi Resolution TEM 2011, with the image analysis software ‘Soft Imaging Systems GmbH analySIS 3.0′. Samples were prepared for analysis by drop-casting a 10 μL aliquot of Pt/C in EtOH, or colloidal Pt nanoparticles, onto carbon-coated copper grids of 3 mm diameter, and dried in air.

Metal loading of Pt/C was determined by atomic absorption spectroscopy (AAS). Pt/C powder was boiled in aqua regia and diluted to 25 mL. The sample was analysed on a Unicam 919 AAS previously calibrated with known Pt standards. The presence of OA at the Pt particle surface was also monitored by FTIR spectra recorded on a PerkinElmer Spectrum One IR spectrometer between 4000–515 cm^−1^.

### Thermo-gravimetric and electrochemical studies

Pt nanoparticles were deposited on Vulcan XC-72R (Pt/C) to prepare electrocatalysts of approximately 15 wt % metal loading. The powder was dried overnight at 60 °C in a vacuum oven. TGA-MS studies were performed on a NETZSCH STA 449 F1, coupled to a quadrupole mass spectrometer Aëolos QMS 403. Pt/C was heated from 35–700 °C at 5 °C min^−1^ in 10 % H_2_ in Argon using a flow rate of 50 mL min^−1^ whilst monitoring ion currents for various *m/z* fragments between 12 and 63.

A catalyst ink was prepared by mixing 2.0 mg of Pt/C catalyst powder with 15 μL of Nafion© dispersion (10 wt %, Sigma Aldrich) in 500 μL Milli-Q water (≥18.2 MΩ). After sonicating to obtain a homogeneous dispersion, a 20 μL aliquot of ink was drop-cast onto a freshly polished glassy carbon working electrode and dried in air. Electrochemical experiments were performed in a two-compartment electrochemical cell using a three electrode set-up, with a Pt wire counter electrode and a KCl-saturated Ag/AgCl reference electrode. The reference electrode in one compartment was connected to the main cell compartment containing the working electrode and counter electrode through a Luggin capillary. The experimental potentials were subsequently converted to the reversible hydrogen reference electrode (RHE), and all potentials quoted herein are given versus this reference, unless otherwise specified. Experiments were performed using an Autolab PGSTAT302N potentiostat, and NOVA software. All solutions were deaerated with high purity argon for at least 15 min before carrying out electrochemical measurements. Each catalyst was electrochemically pre-treated in situ by repeated cycling at 0.500 V s^−1^ between −0.1–1.3 V in 0.5 m H_2_SO_4_ (≥95 %, Sigma Aldrich), until reproducible cyclic voltammograms were obtained. Electrochemical oxidation of formic acid and CO_ads_ procedures were carried out following the previously reported protocols.^9^

## Supporting Information

As a service to our authors and readers, this journal provides supporting information supplied by the authors. Such materials are peer reviewed and may be re-organized for online delivery, but are not copy-edited or typeset. Technical support issues arising from supporting information (other than missing files) should be addressed to the authors.

miscellaneous_information
