# Supplementary material for: Surface Activation of Pt Nanoparticles Synthesised by “Hot Injection” in the Presence of Oleylamine
Source: Chemistry. 2015 Jul 17;21(36):12694–701. doi: 10.1002/chem.201501496 (PMC4676288; doi:10.1002/chem.201501496)
Supplement: Supplementary file 1 [file chem0021-12694-sd1.pdf]

CHEMISTRY
A European Journal

Supporting Information

Surface Activation of Pt Nanoparticles Synthesised by “Hot Injection” in the Presence of Oleylamine

Jo J. L. Humphrey,[a] Sajanikumari Sadasivan,[b] Daniela Plana,[a] Verónica Celorrio,[a] Robert A. Tooze,[b] and David J. Fermín*[a]

chem_201501496_sm_miscellaneous_information.pdf

Table S1: Electrochemical surface area (ECSA) and specific surface areas of Pt/C catalyst (2.24 wt%) following chemical and thermal pretreatments, as determined by CO_{ads} oxidation. The charge associated with the oxidation of a CO monolayer was taken as $Q_{co} = 420$ µC cm^{-2}.

Sample	Area / cm^{-2}	Specific Area / cm^2 mg^{-1}
**Pt/C-Untreated**	0.00	0
**Pt/C-Pyridine**	0.00	0
**Pt/C-EtOH**	0.91	523
**Pt/C-AcOH**	0.10	57
**Pt/C-Air185**	0.34	195
**Pt/C-Ar250**	0.85	489
**Pt/C-Ar350**	0.98	563
**Pt/C-Ar400**	1.46	839
